# Exposure to vector-borne pathogens in candidate blood donor and free-roaming dogs of northeast Italy

**DOI:** 10.1186/s13071-016-1639-6

**Published:** 2016-06-29

**Authors:** Marta Vascellari, Silvia Ravagnan, Antonio Carminato, Stefania Cazzin, Erika Carli, Graziana Da Rold, Laura Lucchese, Alda Natale, Domenico Otranto, Gioia Capelli

**Affiliations:** Istituto Zooprofilattico Sperimentale delle Venezie, Legnaro, Padova, Italy; Dipartimento di Medicina Veterinaria, Università degli Studi di Bari, Valenzano, Italy

**Keywords:** Vector-borne pathogens, Canine blood donors, Free-roaming dogs, Exposure, IFAT, Real-time PCR, Italy

## Abstract

**Background:**

Many vector-borne pathogens including viruses, bacteria, protozoa and nematodes occur in northeast Italy, representing a potential threat to animal and human populations. Little information is available on the circulation of the above vector-borne pathogens in dogs. This work aims to (i) assess exposure to and circulation of pathogens transmitted to dogs in northeast Italy by ticks, sandflies, and mosquitoes, and (ii) drive blood donor screening at the newly established canine blood bank of the *Istituto Zooprofilattico Sperimentale delle Venezie*.

**Methods:**

Blood samples from 150 privately-owned canine candidate blood donors and 338 free-roaming dogs were screened by serology (IFA for *Leishmania infantum, Ehrlichia canis, Anaplasma phagocythophilum, Babesia canis, Rickettsia conorii, R. rickettsii*), microscopic blood smear examination, and blood filtration for *Dirofilaria* spp. All candidate donors and seropositive free-roaming dogs were tested by PCR for *L. infantum*, *E. canis, A. phagocythophilum*, *Babesia/Theileria* and *Rickettsia* spp. The dogs had no clinical signs at the time of sampling.

**Results:**

Overall, 40 candidate donors (26.7 %) and 108 free-roaming dogs (32 %) were seroreactive to at least one vector-borne pathogen. Seroprevalence in candidate donors *vs* free-roaming dogs was: *Leishmania infantum* 6.7 *vs* 7.1 %; *Anaplasma phagocytophilum* 4.7 *vs* 3.3 %; *Babesia canis* 1.3 *vs* 2.7 %; *Ehrlichia canis* none *vs* 0.9 %; *Rickettsia conorii* 16 *vs* 21.3 % and *R. rickettsii* 11 *vs* 14.3 %*.* Seroreactivity to *R. rickettsii,* which is not reported in Italy, is likely a cross-reaction with other rickettsiae. Filariae, as *Dirofilaria immitis* (*n* = 19) and *D. repens* (*n* = 2), were identified in free-roaming dogs only. No significant differences were observed between candidate donors and free-roaming dogs either in the overall seroprevalence of vector-borne pathogens or for each individual pathogen. All PCRs and smears performed on blood were negative.

**Conclusions:**

This study demonstrated that dogs are considerably exposed to vector-borne pathogens in northeast Italy. Although the dog owners reported regularly using ectoparasiticides against fleas and ticks, their dogs had similar exposure to vector-borne pathogens as free-roaming dogs. This prompts the need to improve owner education on the use of insecticidal and repellent compounds in order to reduce the risk of arthropod bites and exposure to vector-borne pathogens. Based on the absence of pathogens circulating in the blood of healthy dogs, the risk of transmission of these pathogens by blood transfusion seems to be low, depending also on the sensitivity of the tests used for screening.

## Background

Several vector-borne pathogens (VBPs), transmitted by ticks, mosquitoes and sandflies, occur in dogs living in northeast Italy. Infection and/or disease are caused by members of the genera *Anaplasma*, *Babesia*, *Borrelia*, *Dirofilaria*, *Ehrlichia*, *Leishmania*, and *Rickettsia* [1–7]. Some of these infections can be life-threatening in dogs (leishmaniosis, cardiopulmonary filariosis, babesiosis) and, in some cases, in humans (leishmaniosis, dirofilariosis, anaplasmosis) [6].

The occurrence of a VBP in a given area is directly dependent on the presence of reservoir hosts and the density of the vectors. For example, the distribution of arthropod vectors in northeast Italy is well known as regards mosquitoes due to the presence of surveillance programs for West Nile virus [8, 9] and other arboviruses transmitted by the tiger mosquitoes, *Aedes albopictus* [10, 11]. *Culex pipiens, Ae. albopictus, Ochlerotatus caspius* and *Ae. vexans* have been identified as the most widespread mosquitoes in the area, including the novel invasive species, *Aedes koreicus,* which has recently been detected [12] and is expanding [13, 14]. All of the above mosquito species have been proven or are suspected to be vectors of *Dirofilaria* spp. [15–18].

*Ixodes ricinus* is the most widespread tick species in northeast Italy and has repeatedly been found to be infected with VBPs that can also affect dogs, i.e. *Borrelia burgdorferi* (*s.l.*), *Rickettsia helvetica, R. monacensis, Anaplasma phagocytophilum, Candidatus Neoehrlichia mikurensis*, *Babesia* spp*.* [19–24]. However, the most common tick species removed from dogs of north Italy is *Rhipicephalus sanguineus,* followed by *I. hexagonus*, *I. ricinus* and *Dermacentor marginatus* [21, 25, 26]. No studies are available on the vectorial role of *Rh. sanguineus* in north Italy.

Two species of sandfly, *Phlebotomus perniciosus* and *P. neglectus,* have been reported in northeast Italy [4, 27, 28]**,** where they are probably responsible for the transmission of *L. infantum* to dogs.

Several VBPs can also be transmitted by blood transfusion. The safety of donated blood with respect to VBPs is guaranteed by serological and molecular screening of dog donors. The Consensus Statements of the American College of Veterinary Internal Medicine (ACVIM) for blood transfusion [29] recommend that donors be screened for VBPs in accordance with the following criteria: (i) the agent is known to be present in the territory; (ii) the agent is known to be potentially transmitted by blood transfusion; (iii) the agent is capable of causing subclinical infection in candidate blood donors; (iv) the disease in the recipient is severe or difficult to foresee. Hence knowledge of VBP circulation among dog populations is pivotal to estimating the risk of transmission by transfusion.

In this study, we assessed exposure to and circulation of pathogens transmitted by ticks, sandflies and mosquitoes to dogs in northeast Italy, including free-roaming dogs and candidate blood donors at the newly established canine Blood Bank of the *Istituto Zooprofilattico Sperimentale delle Venezie.*

## Methods

### Dogs

From January 2014 to December 2015, a total of 488 dogs, including candidate blood donors (CBD) (*n* = 150) and free-roaming dogs (FRD) (*n* = 338), were sampled in several municipalities of the provinces of north and northeast Italy (municipalities of Padua, Treviso, Verona, Venice, Milan and Bologna).

The breed, age, gender and location of the dogs are reported in Table 6.

CBDs were privately-owned dogs and included animals (*n* = 41) from two dog breeders. According to Italian Ministry of Health guidelines (http://tinyurl.com/h7vs3lz), candidate donor dogs need to fulfil the following inclusion criteria: age 2–8 years, body weight ≥ 25 kg, clinically healthy, regularly vaccinated and protected against endo- and ectoparasites.

The FRDs included dogs with no identification (microchip) and privately-owned dogs allowed to wander around, especially in peri-urban and rural areas. According to the Italian law on Companion Animals and the Prevention of Stray Animals (Act no. 281/1991), FRDs are captured by the Local Veterinary Service, housed in shelters and sampled soon after capture. Conversely, CBDs were sampled at their first clinical visit. Whole blood and sera were tested by serology, PCR, blood smear examination, and blood filtration.

### Ethical statement

Informed consent was obtained from the owners of CBDs, as required by the Blood Bank to become eligible donors. The donor screening programme included the collection of information on the health history of the dogs and infectious disease testing. FRDs were sampled and underwent a clinical evaluation by the Local Veterinary Health units at the time of entry to the shelter, as part of the zoonotic agent control programme.

### Diagnostic procedures

#### Serology

Dog sera were tested by means of indirect immunofluorescence assay (IFA) to detect and quantify IgG antibodies against *L. infantum, E. canis, A. phagocythophilum, B. canis, R. conorii* and *R. rickettsii.* The latter pathogen is not reported in the Old World but was included in the screening battery in an attempt to detect cross-reactions with rickettsiae other than *R. conorii*. The in-house assay for *L. infantum* was performed according to the procedure described in the OIE Terrestrial Manual [30]. The serum screening dilution was set at 1:40.

The detection of IgG antibodies against the other pathogens was carried out using commercial kits following the manufacturers’ instructions. The kits were: the *Ehrlichia canis* Canine IFA IgG Kit (Fuller Laboratories, Fullerton, California, USA), serum screening dilution 1:50; the Canine Granulocytic Anaplasmosis IgG IFA Kit (Fuller Laboratories, Fullerton, California, USA), serum screening dilution 1:80; Fluo *Babesia canis* (Agrolabo S.p.A., Scarmagno, Italy), serum screening dilution 1:16; the *Rickettsia conorii* Canine IFA IgG Kit, and the *Rickettsia rickettsii* Canine IFA IgG Kit (Fuller Laboratories, Fullerton, California, USA), serum screening dilution 1:64. Positive and negative controls were added to each slide of the in-house and commercial kits. Two-fold serial dilutions were prepared and tested to define the serum titre of samples testing positive at screening.

### Molecular analyses

DNA was extracted from EDTA-blood samples using a DNeasy Blood & Tissue kit (Qiagen, Valencia, CA, USA), according to the manufacturer’s instructions. The samples were screened for *Babesia/Theileria* spp., *Rickettsia* spp., *Leishmania* spp. and *Ehrlichia canis*, using in-house SYBR Green Real-Time PCR (rPCR) assays, performed with the primers from [31–34] (Table 1). The reactions were carried out in a total volume of 20 μl, containing 10 μl of QuantiFast SYBR Green PCR Master mix 2X (Qiagen GmbH, Germany), sense and reverse primer (concentration reported in Table 1) and 3 μl of extracted DNA. Amplifications were performed in a StepOnePlus™ instrument (Applied Biosystems, Foster City, CA). The thermal profile consisted of 5 min of activation at 95 °C, followed by 40 cycles at 95 °C for 15 s (denaturation), specific annealing temperature (Table 1) for 30 s (annealing) and 60 °C for 30 s (extension). Following amplification, a melting curve analysis was performed by slowly raising the temperature of the thermal chamber from 60 °C to 95 °C to distinguish between specific amplicons and non-specific amplification products. *Anaplasma phagocytophilum* DNA was amplified by conventional PCR targeting the major surface protein gene (msp2), as described elsewhere [35]. To ensure the effectiveness of the nucleic acid extraction, a PCR targeting the 18S rRNA gene internal control was applied [36]. Negative (sterile water) and positive controls (DNA of *Theileria orientalis, Rickettsia felis, Leishmania infantum, Ehrlichia canis* and *Anaplasma phagocytophilum*) were included in each run.

**Table 1:** Target genes and rPCR primers used in this study for pathogen identification

Target organism	Gene	Primer	Sequence (5'-3')	Pc (μM)	TA (°C)	Reference
*Babesia/Theileria*	18S rRNA	BJ1	GTCTTGTAATTGGAATGATGG	0.1	60	[31]
		BN2	TAGTTTATGGTTAGGACTACG	0.1		
*Rickettsia* spp.	rompB	RompB OFm	GTAACCGGAARTAATCGTTTCGT	0.1	58	[32]
		RompB ORm	GCTTTATAACCAGCTAAACCRCC	0.1		
*Leishmania* spp.	COII	COII F	GGCATAAATCCATGTAAGA	0.3	52	[33]
		COII R	TGGCTTTTATATTATCATTTT	0.3		
*Ehrlichia canis*	16S rRNA	CANIS	CAATTATTTATAGCCTCTGGCTATAGGA	0.5	60	[34]
		CA1UR	GAGTTTGCCGGGACTTCTTCT	0.5		

*Abbreviations*: *TA* temperature of annealing; *Pc* primer concentration

16S rRNA = gene coding 16S ribosomal RNA; rompB = Rickettsial Outer Membrane Protein B gene; COII = Cytochrome *c*  oxidase subunit II gene ; 18S rRNA = gene coding 18S ribosomal RNA

### Blood smear examination

The blood smears were stained (Diff Quick Stain Set, Medion Diagnostic AG, Duedingen, SZ) and examined for the presence of any pathogens, i.e. *L. infantum, Babesia spp., Hepatozoon canis, A. phagocytophilum, A. platys*, *E. canis* and microfilariae.

### Filariae screening and identification

One ml of blood in ethylene diamine tetraacetic acid (EDTA) was tested by standard filtration test and staining. The number of microfilariae per millilitre (mf/ml) was calculated as the average of ten counts serially performed on 10 μl of blood samples. The identification of microfilariae was based on their morphology and morphometry [37]. The samples suspected to be positive for *D. repens* were confirmed by PCR and sequencing [38].

### Statistical analysis

The differences between CBDs and FRDs in the prevalence of VBPs for each pathogen and each data element collected on the dogs (breed, gender, age and location) were analysed by the Chi-square test or Fisher’s exact test, where appropriate. Significance was set at *P* < 0.01. The agreement between serological status for *R. conorii/R. rickettsii* and for *A. phagocytophilum/E. canis* was tested using the kappa coefficient [39]. SPSS for Windows, version 13.0 software was used.

## Results

None of the dogs had any clinical signs of VBDs at the time of sampling. The owners of CBDs reported regularly using compounds to control fleas and ticks and taking preventative measures against filariae, while no information was available for FRDs.

Overall, 40 CBDs (26.7 %) and 108 FRDs (32.0 %) were seroreactive to at least one VBP, as shown in Table 2. No significant differences were observed between CBDs and FRDs in the overall seroprevalence of VBPs or for each individual pathogen.

**Table 2:** Serological results and positivity for filariae in candidate blood donors and free-roaming dogs of northeastern Italy, 2014-2015

Pathogens	Candidate blood donors			Free-roaming dogs		
	No. examined	No. positive	%	No. examined	No. positive	%
*Anaplasma phagocytophilum*	150	7	4.7	338	11	3.3
*Babesia canis*	104	0	0	338	3	0.9
*Ehrlichia canis*	150	2	1.3	338	9	2.7
*Leishmania infantum*	150	10	6.7	336	24	7.1
*Rickettsia conorii*	150	24	16.0	338	72	21.3
*Rickettsia rickettsii*	109	12	11.0	336	48	14.3
*R. conorii* and *R. rickettsii*	109	12	11.0	336	33	9.82
*Dirofilaria* spp.	150	0	0	330	21	6.4

Seropositivity was most frequently detected against rickettsiae (26 % in CBDs *vs* 24.3 % in FRDs), followed by *L. infantum* (~7 % in both groups).

Twenty-six of the CBDs (17.3 %) were seroreactive to one test only, 13 (8.7 %) to two and 1 (0.7 %) to three (details in Table 3). Of the FRDs, 56 dogs (16.6 %) were seroreactive to one test only, 43 (12.7 %) to two, 7 (2.1 %) to three and 1 (0.3 %) to four (details in Table 4).

**Table 3:** Candidate blood donors seroreactive to several antigens (*n* = 40)

Candidate donor no.	*Ap*	*Bc*	*Ec*	*Li*	*Rc*	*Rr*
1	0	ne	0	1:40	0	ne
2	0	0	0	1:40	1:64	0
3	1:80	0	0	0	0	0
4	0	0	0	1:80	0	0
5	1:80	0	0	0	0	0
6	0	0	0	0	1:64	0
7	0	ne	0	1:40	0	ne
8	1:160	0	0	0	0	0
9	1:160	0	0	0	0	0
10	0	ne	0	0	1:64	ne
11	1:80	ne	0	0	0	ne
12	0	ne	0	0	1:512	ne
13	0	0	0	0	1:128	0
14	0	0	0	0	1:64	1:64
15	1:80	0	0	0	1:512	1:512
16	0	0	0	0	1:128	0
17	0	0	0	0	1:64	1:64
18	0	ne	0	1:40	0	0
19	0	ne	0	0	1:64	ne
20	0	0	0	0	1:256	1:256
21	0	0	0	0	1:64	1:64
22	0	ne	0	1:40	0	ne
23	0	ne	0	1:40	0	ne
24	0	ne	0	1:40	0	ne
25	0	0	0	1:1280	1:256	0
26	0	ne	0	0	1:256	ne
27	0	0	0	0	1:64	1:64
28	0	0	0	0	1:64	0
29	0	0	0	0	1:64	0
30	0	0	0	0	1:64	1:64
31	0	0	0	0	1:64	1:64
32	0	0	0	0	1:8192	1:2048
33	0	0	0	0	1:64	1:64
34	0	0	1:80	0	0	0
35	0	ne	1:50	0	0	ne
36	0	0	0	0	1:64	0
37	0	0	0	0	1:128	1:128
38	0	ne	0	1:40	0	ne
39	1:80	0	0	0	0	0
40	0	0	0	0	1:128	1:256

*Abbreviations*: *Ap*, *Anaplasma phagocytophilum*; *Bc*, *Babesia canis*; *Ec*, *Ehrlichia canis*; *Li*, *Leishmania infantum*; *Rc*, *Rickettsia conorii*; *Rr*, *Rickettsia rickettsii*; *ne*, not examined

**Table 4:** Free-roaming dogs seroreactive to several antigens (*n* = 108)

Free-roaming dog no.	*Ap*	*Bc*	*Ec*	*Li*	*Rc*	*Rr*
1	0	0	0	0	1:128	1:128
2	0	0	0	0	1:64	0
3	0	0	0	1:40	0	0
4	0	0	0	0	1:128	1:128
5	0	0	0	0	1:128	0
6	0	0	1:50	0	0	0
7	0	0	1:50	0	0	0
8	0	0	0	0	1:64	0
9	0	0	0	0	1:256	1:256
10	0	0	0	0	1:64	0
11	0	0	1:50	0	0	0
12	0	0	1:50	0	1:128	0
13	0	0	0	0	1:64	0
14	0	0	0	0	1:128	0
15	0	0	0	0	1:128	0
16	0	0	0	0	1:64	0
17	0	0	0	0	1:512	1:128
18	0	0	0	1:40	1:128	0
19	0	0	0	1:40	1:256	0
20	0	0	0	0	0	1:64
21	0	0	0	0	1:64	0
22	0	0	0	0	1:128	0
23	0	0	1:50	0	0	0
24	0	0	0	0	0	1:64
25	0	0	0	0	1:64	1:64
26	0	0	0	0	1:64	1:64
27	0	0	0	0	1:64	0
28	0	0	0	0	1:64	1:64
29	0	0	1:50	0	1:64	1:64
30	0	0	1:400	0	0	0
31	0	0	0	0	1:512	1:256
32	0	0	0	0	1:2048	1:2048
33	1:80	0	0	1:40	1:1024	1:512
34	0	0	0	1:40	0	0
35	0	0	0	1:40	1:64	0
36	0	0	0	0	1:128	0
37	0	0	0	0	1:128	1:128
38	0	0	0	1:40	1:1024	1:256
39	1:80	0	0	1:40	0	0
40	0	0	0	0	1:64	0
41	0	0	0	0	1:128	0
42	0	0	0	0	1:128	0
43	0	0	0	0	1:64	0
44	0	0	0	1:40	1:256	1:1280
45	0	0	0	0	1:64	1:64
46	0	0	0	1:40	0	0
47	0	0	0	0	1:64	0
48	0	0	0	0	1:128	0
49	1:160	0	0	0	0	0
50	1:320	0	0	ne	1:64	ne
51	0	0	0	ne	1:256	ne
52	0	0	0	0	1:64	1:64
53	0	0	0	0	1:64	1:64
54	0	0	0	1:40	0	0
55	1:160	0	0	1:40	1:64	0
56	0	0	0	1:40	0	0
57	0	0	0	0	0	1:64
58	1:160	0	0	0	1:256	1:256
59	1:320	0	0	1:40	0	0
60	0	0	0	1:40	0	0
61	0	0	0	0	1:64	0
62	0	0	0	0	1:64	0
63	0	1:16	0	0	1:256	0
64	1:80	0	0	0	1:64	0
65	0	0	0	0	0	1:1024
66	0	0	0	0	1:1024	1:2048
67	0	0	0	0	1:1024	1:2048
68	0	0	0	0	1:256	1:256
69	0	0	0	1:40	0	0
70	0	0	0	1:40	0	0
71	0	0	0	1:40	1:128	1:64
72	0	0	0	1:40	1:128	0
73	0	0	0	0	1:128	1:64
74	0	0	0	0	1:256	1:64
75	0	0	0	0	1:128	0
76	1:80	0	0	0	0	1:64
77	0	0	0	0	0	1:64
78	0	0	0	0	1:64	1:256
79	0	0	0	0	1:64	0
80	0	0	0	0	1:64	1:64
81	0	0	0	1:40	0	1:64
82	1:80	0	0	0	1:256	1:512
83	0	0	0	0	1:256	1:512
84	0	0	0	0	1:1024	0
85	0	0	0	0	1:64	0
86	0	0	0	0	1:64	0
87	0	0	0	0	0	1:128
88	0	0	0	0	1:256	1:128
89	0	0	0	0	0	1:64
90	0	0	0	0	1:128	1:128
91	0	0	1:50	0	0	0
92	0	0	0	0	1:64	1:256
93	0	0	1:100	0	0	0
94	0	0	0	0	0	1:64
95	0	0	0	0	0	1:64
96	1:5120	0	0	0	0	0
97	0	0	0	0	1:512	1:1024
98	0	0	0	1:40	0	0
99	0	1:16	0	0	0	0
100	0	1:16	0	0	0	0
101	0	0	0	1:40	0	0
102	0	0	0	0	1:64	1:64
103	0	0	0	0	1:64	1:256
104	0	0	0	0	1:64	1:128
105	0	0	0	1:40	0	0
106	0	0	0	0	1:128	1:128
107	0	0	0	0	1:64	1:64
108	0	0	0	1:40	0	0

*Abbreviations*:* Ap*, *Anaplasma phagocytophilum*; *Bc*, *Babesia canis*; *Ec*, *Ehrlichia canis*; *Li*, *Leishmania infantum*; *Rc*, *Rickettsia conorii*;* Rr*, *Rickettsia rickettsii*; *ne*, not examined

The most common seropositivity for two antigens in the same dog (co-reaction) was for *R. conorii* and *R. rickettsii,* in both groups of dogs (Tables 3 and 4). Specifically, of the 101 dogs testing positive for *Rickettsia* spp., 49 (48.5 %) were positive for both rickettsiae, 41 (40.6 %) exclusively for *R. conorii* and 11 (10.9 %) exclusively for *R. rickettsii*, the latter all being FRDs. Agreement between seropositivity for the two rickettsiae was moderate (k = 0.586), suggesting a certain degree of cross-reactivity. Conversely, seroreactivity for *A. phagocytophilum* and *E. canis* was completely discordant (k = -0.29), suggesting that these antigens did not cross-react in our test.

The distribution of antibody titres was similar between CBDs and FRDs (*P* > 0.05) (Table 5).

**Table 5:** Distribution of antibody titres for each pathogen in candidate blood donors and free-roaming dogs in northeast Italy, 2014–2015

Pathogens^a^	Candidate blood donors				Free-roaming dogs			
	Pos 1^st^ dilution	Pos 2^nd^ dilution	Pos > 2^nd^ dilution	Max. titre	Pos 1^st^ dilution	Pos 2^nd^ dilution	Pos > 2^nd^ dilution	Max. titre
*Anaplasma phagocytophilum*	5	2	0	1:160	5	3	3	1:5120
*Babesia canis*	0	0	0	–	3	0	0	1:16
*Ehrlichia canis*	1	1	0	1:100	7	1	1	1:400
*Leishmania infantum*	8	1	1	1:1280	24	0	0	1:40
*Rickettsia conorii*	14	4	6	1:8192	33	19	20	1:2048
*Rickettsia rickettsii*	7	1	4	1:2048	22	9	17	1:2048

^a^ Serum screening dilution: *Ap* (1:80); *Bc* (1:16); *Ec* (1:50); *Li* (1:40); *Rc* (1:64); *Rr* (1:64)

Among the dogs testing positive for *L. infantum*, only one dog showed a high antibody titre, consistent with clinical leishmaniosis (1:1280). The antibody titre for *R. conorii* or *R. rickettsii* was above 1: 320 (Table 5) in a total of 26 dogs (5.3 %). The 12 CBDs testing positive to both species of *Rickettsia* showed very similar titres. Conversely, 10 (27 %) of the 37 FRDs presenting co-reactions had a higher titre for *R. rickettsii*.

No significant differences were found between seropositivity and the data given in Table 6.

**Table 6:** Breed, age, gender and location of the dogs according to seropositivity

Data	*A. phagocythophilum*	*B. canis*	*E. canis*	*L. infantum*	*R. conorii*	*R. rickettsii*
	pos/tested (%)	pos/tested (%)	pos/tested (%)	pos/tested (%)	pos/tested (%)	pos/tested (%)
Breed						
crossbreed	10/327 (3.1)	3/323 (0.9)	9/327 (2.8)	23/327 (7.0)	68/327 (20.8)	46/323 (14.2)
other breed	7/153 (4.6)	0/111 (0)	2/153 (1.3)	11/153 (7.2)	25/153 (16.3)	13/116 (11.2)
Age (months)						
≤12	2/91 (2.2)	1/91 (1.1)	1/91 (1.1)	9/91 (9.9)	16/91 (17.6)	10/91 (11.0)
>12 ≤ 36	5/88 (5.7)	1/72 (1.4)	0/87 (0)	11/87 (12.6)	15/87 (17.2)	7/74 (9.5)
>36 ≤ 60	3/69 (4.3)	1/59 (1.7)	1/70 (1.4)	3/69 (4.3)	9/69 (13.0)	8/59 (13.6)
> 60	4/68 (5.9)	0/61 (0)	0/69 (0)	2/69 (2.9)	14/69 (20.3)	14/63 (22.2)
Gender						
female	11/177 (6.2)	1/148 (0.7)	3/177 (1.7)	13/176 (7.4)	30/176 (17.0)	16/150 (10.7)
male	6/303 (2.0)	2/286 (0.7)	8/304 (2.6)	21/304 (6.9)	64/304 (21.1)	44/289 (15.2)
Location						
Padua	9/226 (4.0)	3/225 (1.3)	2/228 (0.9)	16/226 (7.1)	41/227 (18.1)	34/223 (15.2)
Treviso	2/157 (1.3)	0/140 (0)	7/158 (4.4)	10/157 (6.4)	39/157 (24.8)	18/140 (12.9)
Venice	3/59 (5,1)	0/43 (0)	0/59 (0)	4/59 (6.8)	8/59 (13.6)	5/48 (10.4)
Other	4/46 (8.7)	0/34 (0)	2/46 (4.3)	4/46 (8.7)	8/46 (17.4)	3/34 (8.8)

Microfilariae were found exclusively in FRDs (*n* = 21; 6.4 %) (*χ*^*2*^ = 9.982, *df* = 1, *P* = 0.0016) and were identified as *D. immitis* (*n* = 19) and *D. repens* (*n* = 2). All dogs testing positive for microfilariae were from the province of Padua (21/219; 9.6 %). The microfilariae of *D. immitis* per ml of blood (mf/ml) ranged from 4 to 26,620 (mean = 413), and numbered 26 and 14,440 mf/ml, respectively, in the two dogs found positive for *D. repens*.

All PCRs and smears performed on blood were negative.

## Discussion

This study has demonstrated that dogs are considerably exposed to VBPs in northeast Italy. The most frequent pathogens encountered by dogs in this area are members of the genus *Rickettsia*. Considering that *R. rickettsii* (the agent of Rocky Mountain Spotted Fever) is not reported in the Old World, the seroreactivity to this pathogen in the dogs in our study was the effect of a cross-reaction with other rickettsiae, as reported elsewhere [41] and stated in the instructions accompanying the serological kit used. The following species of *Rickettsia* were detected in north Italy: *R. helvetica* and *R. monacensis*, a common finding in *Ixodes ricinus* ticks [19, 20, 24], and *R. slovaca* and *R. raoultii,* detected in *Dermacentor marginatus* ticks removed from wild boars [42]. The circulation of many other *Rickettsia* spp. is reported in hosts and vectors in central and southern Italy, the most common being *R. massiliae*, *R. aeschlimannii* and *R. conorii israeliensis* [25, 43, 44].

*Rickettsia conorii* (the agent of the Mediterranean Spotted Fever)*,* has been detected almost exclusively in southern Italy, in both humans [40] and dogs [2]. It can therefore be argued that a certain level of seroreactivity to this antigen is due to a cross-reaction with other *Rickettsia* spp. 

The high rate of exposure to rickettsiae and the low rate of exposure to *A. phagocytophilum* of the dogs in our study is consistent with the rate of infection found in *Ixodes ricinus* in the same area between 2005 and 2008 (i.e. 13.1 % and 3.7 % for *R. helvetica* and *R. monacensis*, respectively, and 1.5 % for *A. phagocytophilum*) [24].

In our survey, three FRDs had high titres for A*. phagocytophilum*, without any evident clinical signs, indicating either a previous infection or a subclinical/mild infection in dogs not subjected to laboratory testing to carefully evaluate their clinical status. However, in a previous study using an IFA test, seroprevalence was not significantly different between sick (47 %) and healthy dogs (40 %) [45].

Our dogs were found to have a very low rate of exposure to other pathogens transmitted by *Rhipicephalus* ticks (*Ehrlichia* and *Babesia*) compared to studies performed in central [46] and southern Italy [47, 48], which reported seroprevalence to be up to 46 % for *E. canis* and as high as 70 % for *Babesia* spp. This is likely due to the lower abundance of the brown dog tick of the *Rh. sanguineus* group in the northern compared to southern Italy, where warmer temperatures throughout the year may contribute to increasing tick abundance [49].

In addition, many studies have suggested that vector competence of different populations (haplotypes or sibling species) of the *R. sanguineus* group may vary, reviewed in [50]. However, populations of *R. sanguineus* have never been accurately mapped in northern Italy.

The second pathogen to which FRDs and CBD dogs are exposed is *L. infantum*. All but one of the animals showed a serological titre below 1:80, a cut-off not usually indicative of infection [51] and thus requiring confirmation by other tests or seroconversion. The IFA used in this study showed no [52], or a very low rate of, cross-reaction with other VBPs [53], suggesting that the seroreactivity is most likely due to contact with an infected sandfly.

This is consistent with the history of *Leishmania* in northern regions. Indeed, 20 years ago canine leishmaniosis was regarded as an infection “imported” from endemic areas of the south. The scenario has quickly changed [5], with new foci continuing to emerge in northern regions [4, 54, 55] and phlebotomine vectors recently being found in the northernmost part of the eastern Italian Alps [27].

Detection of *D. immitis* and *D. repens* in FRDs indicate that both nematodes are still circulating in the area of investigation, particularly in the lowlands around Padua. Northeastern Italy is an endemic area for *D. immitis,* with prevalences of up to 80 % being reported in the past [5, 56]. Surveys performed in the 1990s in the same province found 67 % of 175 stray dogs at the local municipality shelter to be infected by *D. immitis* [57]. At the end of the 1990s, contact between a mosquito infected by *D. immitis* and a host was estimated to occur every four nights for dogs and within two weeks for humans [7]. In subsequent years, after the advent of efficient preventative measures, the prevalence of heartworm infection dramatically decreased, especially in urban areas (unpublished data). However, in rural areas, both *D. immitis* and *D. repens* are still circulating, as demonstrated by the positivity of the FRDs taking part in this study and the presence of infected mosquitoes. The screening in 2010 of over 10,000 mosquitoes captured in the same area as this study revealed the presence of *D. immitis,* alone or in combination with *D. repens,* in 13 and two of the 20 monitored sites, respectively [17].

Despite the considerable rate of exposure to VBPs, none of the study dogs presented evident clinical signs and/or circulating pathogens at the time of sampling. This is not surprising, since the detection of pathogens in the bloodstream can be difficult even in clinical cases [1, 2, 41, 59–61]**.** A study performed on 650 sick dogs, yielded positive PCR results for *Rickettsia* spp. in 0.4, 1.4 and 3.3 % of dogs from northern, central and southern Italy, respectively [2]. Another study was unable to find *A. phagocytophilum* and *Rickettsia* spp. in 135 sick Italian dogs and found a low prevalence of *E. canis* (1.8 %) in 54 dogs in the north [58].

Conversely, in dogs showing clinical signs consistent with babesiosis, the pathogen is often detected both by blood smear examination and PCR [60, 61]. In north Italy, *Babesia canis* was found by PCR in 30/103 sick dogs (29 %) and *B. vogeli* in 1/103 (0.9 %) [61]. However, 55 % of the dogs infected by *B. canis* had travelled in eastern Europe, where babesiosis and the tick vector *Dermacentor reticulatus* are endemic [62], and were therefore likely to be imported cases.

The comparable rate of exposure to pathogens transmitted by ticks and sandflies in CBDs and FRDs was unexpected considering that CBD owners are very careful about their dogs’ health, including the control of ectoparasites and prevention of dirofilariosis. Conversely, less or no care was expected to be taken of FRDs. This assumption is strengthened by the fact that positivity for filariae was found exclusively in FRDs, while the owners of CBDs proved to correctly use prophylactic measures against dirofilariosis. The similar seroprevalence for VBPs in the two groups may be explained by limited or incorrect use of compounds with repellent activity against arthropod vectors, as demonstrated by the results of a questionnaire administered to dog and cat owners in Portugal [63]. Repellents have in fact been proven to prevent vector bites and consequently the transmission of pathogens, even in highly endemic areas of south Italy [47, 64–66].

Our results confirm that serological positivity against tick-borne pathogens, even with very high titres, has to be carefully considered. Clinical observations, sensitive PCRs and repeated serological tests must be applied to confirm acute or chronic infections caused by rickettsial agents [41]. In addition, more specific, commercially available serological rickettsial assays, coupled with deeper knowledge of the pathogenic potential of the different species, are greatly required.

Although exposure to VBPs is frequent for dogs living in northeast Italy, our results suggest that the risk of transmission by blood transfusion is low, if donors are carefully selected. Specifically, in areas endemic for *Ixodes* spp., it may be difficult to identify donors that are seronegative for *Anaplasma* spp. and *Rickettsia* spp. It might, therefore, be acceptable to use seropositive but PCR negative dogs as donors in such situations [67]. Conversely, serological screening for *E. canis* and *L. infantum* remains mandatory, since the antibody titres are predictive of infection. Finally, the diffusion and prevalence of other pathogens, such as *Bartonella* and haemoplasmas, should be investigated, as recommended by the updated Consensus Statements of the American College of Veterinary Internal Medicine (ACVIM) [67].

## Conclusions

This study has improved our knowledge on the circulation of important VBPs in northeast Italy and has demonstrated a considerable rate of exposure to VBPs among dog populations. Although owners of CBDs reported regularly using compounds against fleas and ticks, their dogs had similar exposure to vector-borne pathogens as free-roaming dogs. This prompts the need to improve owner education on the use of repellent compounds in order to prevent arthropod bites and, consequently, the transmission of VBPs. The seroreactivity of CBDs to all the screened VBPs reinforces the need to continue applying this panel of PCRs at each blood donation. The test panel should also be continually revised according to additional information gathered on the introduction of pathogens and/or vectors from endemic areas.

## Abbreviations

VBP, vector-borne pathogen; CBD, candidate blood donors; FRD, free-roaming dogs; ACVIM, American College of Veterinary Internal Medicine; PCR, polymerase chain reaction; EDTA, Ethylene Diamine Tetraacetic Acid; DNA, Deoxyribonucleic acid; rRNA, ribosomal ribonucleic acid; IFA, Immunofluorescence Assay.
